# Omicron B.1.1.529 variant infections associated with severe disease are uncommon in a COVID-19 under-vaccinated, high SARS-CoV-2 seroprevalence population in Malawi

**DOI:** 10.1016/j.eclinm.2022.101800

**Published:** 2022-12-30

**Authors:** Upendo L. Mseka, Jonathan Mandolo, Kenneth Nyoni, Oscar Divala, Dzinkambani Kambalame, Daniel Mapemba, Moses Kamzati, Innocent Chibwe, Marc Y.R. Henrion, Kingsley Manda, Deus Thindwa, Memory Mvula, Bright Odala, Raphael Kamng'ona, Nelson Dzinza, Khuzwayo C. Jere, Nicholas Feasey, Antonia Ho, Abena S. Amoah, Melita Gordon, Todd D. Swarthout, Amelia Crampin, Robert S. Heyderman, Matthew Kagoli, Evelyn Chitsa-Banda, Collins Mitambo, John Phuka, Benson Chilima, Watipaso Kasambara, Kondwani C. Jambo, Annie Chauma-Mwale

**Affiliations:** a Malawi-Liverpool-Wellcome Programme, Blantyre, Malawi; b Public Health Institute of Malawi, Lilongwe, Malawi; c Department of Clinical Sciences, Liverpool School of Tropical Medicine, Liverpool, United Kingdom; d National Statistical Office, Zomba, Malawi; e Institute of Infection, Veterinary and Ecological Sciences, University of Liverpool, Liverpool, United Kingdom; f University of Glasgow, Glasgow, United Kingdom; g London School of Hygiene and Tropical Medicine, London, United Kingdom; h Malawi Epidemiology and Intervention Unit, Lilongwe, Malawi; i NIHR Mucosal Pathogens Research Unit, Research Department of Infection, Division of Infection and Immunity, University College London, London, United Kingdom; j Kamuzu University of Health Sciences (formerly University of Malawi, College of Medicine) Blantyre, Malawi

**Keywords:** Omicron, SARS-CoV-2, Anti-RBD antibodies, COVID-19, Malawi

## Abstract

**Background:**

The B.1.1.529 (Omicron) variant of severe acute respiratory syndrome coronavirus 2 (SARS-CoV-2) has resulted in the fourth COVID-19 pandemic wave across the southern African region, including Malawi. The seroprevalence of SARS-CoV-2 antibodies and their association with epidemiological trends of hospitalisations and deaths are needed to aid locally relevant public health policy decisions.

**Methods:**

We conducted a population-based serosurvey from December 27, 2021 to January 17, 2022, in 7 districts across Malawi to determine the seroprevalence of SARS-CoV-2 antibodies. Serum samples were tested for antibodies against SARS-CoV-2 receptor binding domain using WANTAI SARS-CoV-2 Receptor Binding Domain total antibody commercial enzyme-linked immunosorbent assay (ELISA). We also evaluated COVID-19 epidemiologic trends in Malawi, including cases, hospitalisations and deaths from April 1, 2021 through April 30, 2022, collected using the routine national COVID-19 reporting system. A multivariable logistic regression model was developed to investigate the factors associated with SARS-CoV-2 seropositivity.

**Findings:**

Serum samples were analysed from 4619 participants (57% female; 60% aged 18–50 years), of whom 878/3794 (23%) of vaccine eligible adults had received a single dose of any COVID-19 vaccine. The overall assay-adjusted seroprevalence was 83.7% (95% confidence interval (CI), 79.3%–93.4%). Seroprevalence was lowest among children <13 years of age (66%) and highest among adults 18–50 years of age (82%). Seroprevalence was higher among vaccinated compared to unvaccinated participants (1 dose, 94% vs. 77%, adjusted odds ratio 4.89 [95% CI, 3.43–7.22]; 2 doses, 97% vs. 77%, aOR 6.62 [95% CI, 4.14–11.3]). Urban residents were more likely to be seropositive than those from rural settings (91% vs. 78%, aOR 2.76 [95% CI, 2.16–3.55]). There was at least a two-fold reduction in the proportion of hospitalisations and deaths among the reported cases in the fourth wave compared to the third wave (hospitalisations, 10.7% (95% CI, 10.2–11.3) vs. 4.86% (95% CI, 4.52–5.23), p < 0.0001; deaths, 3.48% (95% CI, 3.18–3.81) vs. 1.15% (95% CI, 1.00–1.34), p < 0.0001).

**Interpretation:**

We report reduction in proportion of hospitalisations and deaths from SARS-CoV-2 infections during the Omicron variant dominated wave in Malawi, in the context of high SARS-CoV-2 seroprevalence and low COVID-19 vaccination coverage. These findings suggest that COVID-19 vaccination policy in high seroprevalence settings may need to be amended from mass campaigns to targeted vaccination of reported at-risk populations.

**Funding:**

Supported by the 10.13039/100000865Bill and Melinda Gates Foundation (INV-039481).

**Research in context**

**Evidence before this study**

Sub-Saharan Africa has poor COVID-19 vaccination coverage but has had a relatively less severe. COVID-19 pandemic to date compared to Europe and America. Early 2022, Madhi SA et al. reported high SARS-CoV-2 seropositivity in South Africa prior to the onset of the Omicron wave, and this was associated with reduced incidence of COVID-19 hospitalisations, recorded deaths and excess deaths; thereafter. Subsequent work in highly vaccinated populations have also shown a similar trend. However, in COVID-19 under-vaccinated populations, data on the same is lacking.

**Added value of this study**

Similar to previous serosurveys in sub-Saharan Africa, SARS-CoV-2 seroprevalence was very high (>80%) in a COVID-19 under-vaccinated population, indicating widespread community transmission than can be deduced from the reported national cases. Consistent with Madhi SA et al.*,* but in a highly under-vaccinated and resource-constrained setting, the high infection-driven seroprevalence was associated with a similar dissociation between COVID-19 cases and severe COVID-19, indicating an attenuation of the pandemic likely due to widespread infection-induced immunity.

**Implications of all the available evidence**

The primary results support amendment of vaccination policy of current COVID-19 vaccines in high seroprevalence resource-constrained settings from mass vaccination campaigns to targeted; vaccination that focuses on reported at-risk populations. However, longevity of infection-induced; immunity against severe COVID-19 disease in these settings needs to be explored to further optimise; the COVID-19 vaccination policies.

## Introduction

The B.1.1.529 (Omicron) variant of severe acute respiratory syndrome coronavirus 2 (SARS-CoV-2) was first reported in South Africa on 25th November 2021. Within approximately 25 days the Omicron variant was responsible for over 90% of the new clinical cases in South Africa, compared to 80% with the Delta variant 100 days after its emergence in the same setting.^1^ Compared to wild type SARS-CoV-2, the Omicron attack rate was four times higher and the Delta rate was twice as high.^2^ Genomic sequencing of the Omicron variant has revealed a wide range of non-synonymous mutations,^3^^,^^4^ with 30 mutations identified within its spike protein that have been linked to its high transmission rate and greater immune evasion of neutralizing antibodies.^5^, ^6^, ^7^

The COVID-19 pandemic trajectory in Malawi has mirrored that of South Africa.^8^ Post emergence of variants of concern in South Africa, Malawi has experienced three COVID-19 waves, caused by Beta, Delta and Omicron.^8^ As of January 2022, the number of reported COVID-19 clinical cases and deaths in Malawi were 84,420 and 2558, respectively, while the vaccination coverage of the eligible population of 18 years and above was 22%.^9^ However, data from a serosurvey among blood donors in Malawi showed very high seropositivity (>65%) by July 2021.^10^ Since seropositivity indicates that someone has been infected in the past/present or has been vaccinated, the high seropositivity suggested widespread community transmission and a huge under-ascertainment of SARS-CoV-2 infections and possibly deaths. A recent sero-epidemiological study in South Africa found high SARS-CoV-2 seropositivity in COVID-19 unvaccinated (68%) and vaccinated (93%) individuals prior to the onset of the Omicron wave, and this was associated with reduced incidence of COVID-19 hospitalisations, recorded deaths and excess deaths thereafter.^1^ Consequentially, South Africa has taken a more pragmatic approach to COVID-19, accepting the limited success in preventing infections and allowing for policy development that is built on minimising severe COVID-19, while balancing the direct and indirect societal effects of COVID-19 ^11^^,^^12^. However, South Africa is the richest economy on the continent,^13^ had stringent lockdowns, and has epidemiological profiles of communicable and non-communicable co-morbidities that differ from elsewhere in the region.^11^^,^^12^^,^^14^ To inform public health policy decisions in very low COVID-19 vaccine coverage and resource-constrained countries such as Malawi, further data are urgently needed.

We, therefore, undertook a population-based serosurvey in Malawi that was conducted from December 27, 2021 to January 17, 2022, during the Omicron-dominant fourth wave. Furthermore, we link this data to the national COVID-19 epidemiologic trends in Malawi, including clinical cases, hospitalisations and deaths, from April 1, 2021 through April 30, 2022. Understanding seroprevalence for SARS-CoV-2 provides information on the burden of COVID-19 in the population and can provide inference on population immunity that is essential for informing public health policy.

## Methods

### Study design and study population

We conducted a cross-sectional survey in 12 districts with urban centers (Blantyre, Lilongwe, Zomba, Mzimba North) and rural districts (Dedza, Machinga, Dowa, Kasungu, Nkhata Bay, Ntcheu, Nsanje, and Neno) from among 29 districts. Malawi has five health zones in three administrative regions (North, Southeast, Southwest, Central East, and Central West). Districts were selected across Malawi using simple randomisation within the health zones, choosing 2 districts per health zone, making a total of 10 districts. Additionally, the two largest cities of Blantyre and Lilongwe were added, totaling 12 districts. The districts of Lilongwe (2,626,901), Zomba (851,737) and Blantyre (1,251,484) have both rural and urban areas.

The survey population included participant aged ≥5 years. Participants ≥18 years of age provided written informed consent, while those aged 12–17 years of age provided assent and consent from a parent/guardian and those under 12 years consent was sought from the parent/guardian.

The National Health Sciences Research Committee (NHSRC) (Protocol #21/02/2671) gave ethical approval for this work and further authorization was also obtained from the District Health Offices of the participating districts. Community sensitization within each district was implemented through community leadership and study staff prior to data collection. All participants provided written informed consent, including assent for children; those who were approached were informed that they were free to decline participation or withdraw at any time without any negative repercussion.

### Sample size calculation

The study used a stratified multistage probability sample design, with strata defined by five health zones, primary sampling units (PSU) defined by enumeration areas (EAs) within strata, second-stage sampling units defined by households within EAs, and finally eligible persons within households.

We therefore assumed 5% prevalence of SARS-CoV-2 antibodies, a margin of error of ±5%, 95% precision of estimates, and a design effect of 1.26, based on an earlier seroprevalence study done in October 2020.^15^ Using the following formula, we calculated a sample size (n) of 7744 persons.

n=DEFFxNp(1−p)d2Z1−α/22×(N−1)+p×(1−p)

where;

Population size (N): 17,563,749

Hypothesized estimate of the prevalence of SARS-CoV-2 (*p*): 5% ± −5

Confidence limits as % of 100 (absolute +/− %) (d): 5%

Design effect (DEFF): 1.26 (*arbitrary*)

The PSUs were selected with probabilities proportionate to the number of households in the EA based on the 2018 census.^16^ The second-stage sampling units were selected from lists of households complied by trained staff for each of the sampled PSUs. Upon completion of the listing process, a random systematic sample of households was selected from each PSU within each health zone to the extent feasible. Within the sampled households, all eligible participants were included in the study sample for data collection. Assuming an average household size of 4.4 from the national census,^16^ the estimated households to be recruited was 1760.

**Fig. 1:**
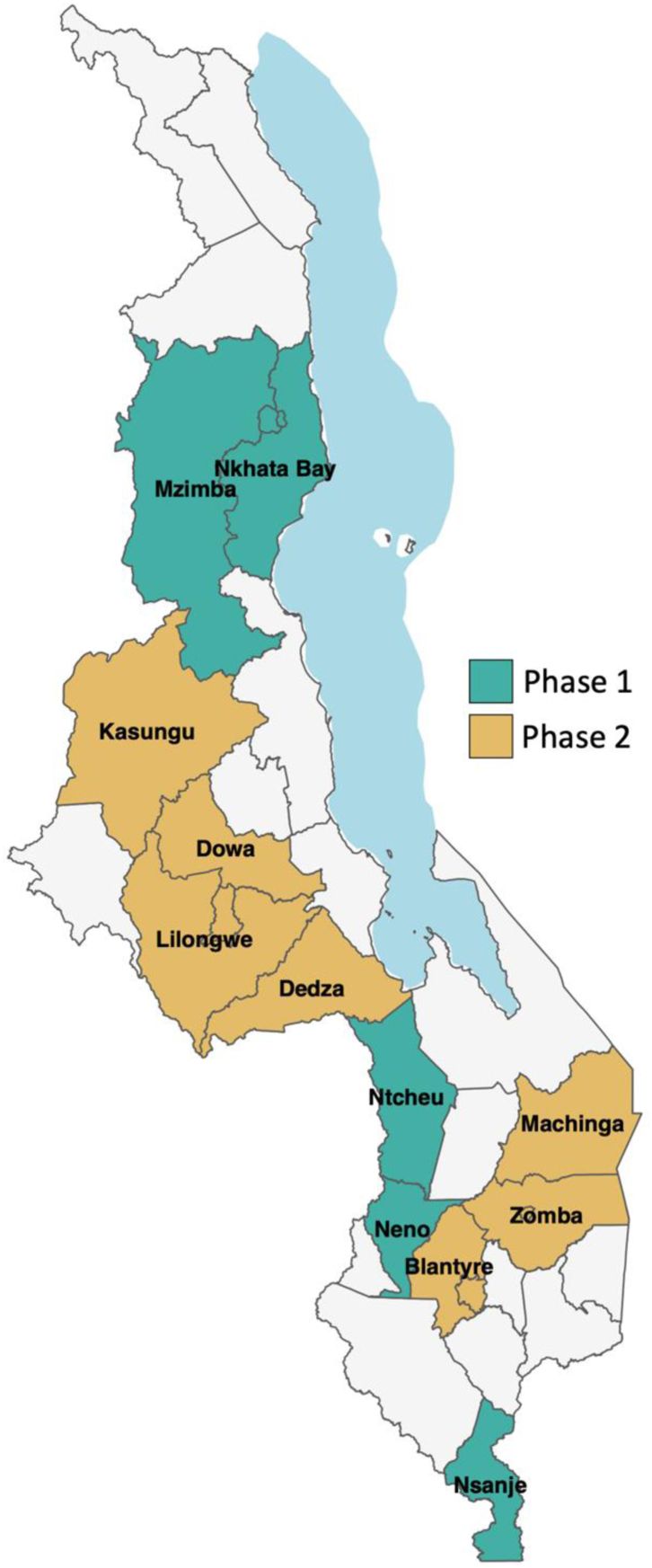
**Study location.** The study was conducted in 7 districts (Phase 2) in Malawi, covering 4 of the 5 health zones. The 7 districts harbor 45% of Malawi's population and also host 3 of 4 Malawi's cities. Lilongwe is largest and also the Capital City, while Blantyre the second largest and the Commercial City. Blantyre has reported the highest number of COVID-19 cases, followed by Lilongwe.

Due to logistical and operational challenges, the study was divided into two Phases. Phase 1 recruited participants in 5 districts (Mzimba North, Neno, Ntcheu, Nkhatabay, Nsanje), while Phase 2 recruited participants in 7 districts (Kasungu, Dowa, Dedza, Lilongwe, Zomba, Machinga, and Blantyre) (Fig. 1). The 5 districts in Phase 1 constitute 13% of the Malawi's population (2,221,932 of 17, 563, 749 persons), while the 7 districts in Phase 2 harbor 45% of the population of Malawi (7,911,594 of 17, 563, 749 persons).^16^ The recruitment for Phase 2 was conducted from 27th December 2021 to 17th January 2022 and forms the basis of this manuscript. The expected number of households for the Phase 2 was 1420 with a total sample size of 6248 persons.

### Data and sample collection

After the consenting process, a standard COVID-19 screening tool was implemented using electronic data capturing (Open Data Kit, ODK), including demographic data, socio-economic status, chronic illnesses (Diabetes Mellitus, Hypertension, Asthma, Chronic Lung Disease, Heart Disease, Chronic kidney disease, Stroke, Tuberculosis, Chronic liver disease, HIV and Cancer), and COVID-19 vaccination status from each enrolled individual. COVID-19 vaccination status was ascertained from all participants using the individual national COVID-19 vaccination card. At the time of the study, the available COVID-19 vaccines were ChAdOx1 nCoV-19 and Ad26.COV2.S.

Peripheral venous blood samples (4 ml) were obtained from all enrolled participants. Samples were transported to the district laboratory, where serum was separated by density centrifugation, aliquoted and kept at 2–8 °C for transportation to the main storage facility at the National Public Health Reference Laboratory (NPHRL) repository. Serum was archived at −80 °C until testing.

### Serological analysis

SARS-CoV-2 Receptor Binding Domain protein immunoglobulin were measured qualitatively in sera using the WANTAI SARS-CoV-2 total antibody commercial ELISA kit as described previously,^10^ following the manufacturer's instructions (Beijing Wantai Biological Pharmacy Enterprise Co., Ltd., China; WS-1096). The results are expressed as a ratio which is calculated by dividing the optical densities of the test sample by those of assay cutoff (mean OD of three internal negative calibrators + 0.16). Specimens giving a ratio of <0.9 were reported as negative for this assay, a ratio of >1.1 were reported as positive, and a ratio between 0.9 and 1.1 were reported as borderline.^10^ Samples with borderline results were retested using a confirmatory assay described below.

As a confirmatory assay, a multiplexed MSD immunoassay (MSD, Rockville, MD) was used to measure anti-SARS-CoV-2 IgG antibodies against Spike, Nucleocapsid and Receptor Binding Domain. A MULTI-SPOT® 96-well, 10 Spot Plate was coated with 7 SARS CoV-2 antigens (RBD, N, Spike Alpha, Spike Beta, Spike Gamma, Spike Delta and Spike Omicron). The plates were blocked with MSD Blocker A following which reference standard, controls and samples diluted 1:500 in diluent buffer were added. After incubation, detection antibody was added (MSD SULFO-TAG™ Anti-Human IgG Antibody) and then MSD GOLD™ Read Buffer B was added and plates read using a MESO® QuickPlex SQ 120 MM Reader.

### National COVID-19 data sources

Data regarding daily cases, hospitalisations, and deaths were sourced from the Public Health Institute of Malawi (PHIM), from 1st April 2021 through 30th April 2022.^17^ In brief, the routine national COVID-19 reporting system works as follows: data from multiple sources at district level covering a 24-h period (6am–6am) are sent to national level through the national Public Health Emergency Operations Centre (PHEOC). The data undergoes a triple verification system against three sources, surveillance, laboratory, and case management, and then released to the public. During the stated time period above, the criteria for COVID-19 testing was suspected cases or close contacts, symptomatic returning travelers, outbound travelers and anyone scheduled for a surgical procedure.

Cases included asymptomatic and symptomatic infections with SARS-CoV-2 confirmed by either a nucleic acid amplification assay or a rapid antigen test. Hospitalisations included admissions for SARS-CoV-2 infection, as well as admissions for other illnesses in which SARS-CoV-2 infection was incidentally identified on routine screening at the time of admission. Death attributable to COVID-19 was defined as a death resulting from a clinically compatible illness in a probable or confirmed COVID-19 case unless there is a clear alternative cause of death that was not related to COVID-19 disease (e.g., trauma), according to Malawi national guidelines.

### Statistical methods

Statistical analyses were performed using R statistical package (version 4.1.0).^18^ Categorical variables were summarized as frequencies and percentages. Seropositivity was calculated as the proportion of those who tested positive among all those tested. The overall seroprevalence of the SARS-CoV-2 antibodies was adjusted for both the assay's sensitivity 29/30 (96.7% [95% CI 85.5–99.9]) and specificity 78/80 (97.5% [95% *CI* 92.5–99.9]),^19^ using bootComb.^20^ The χ^2^ test or Fisher's exact test was used to compute the proportions of participants by number of COVID-19 vaccine doses received across several variables. A multivariable logistic regression model was developed to investigate the factors associated with SARS-CoV-2 seropositivity. The factors included age, sex, occupational exposure and COVID-19 vaccination, as categorical variables, which were chosen based on known predictors of seropositivity. A p-value of <0.05 was considered statistically significant.

### Role of funding

The funders were not involved in the design of the study; in the collection, analysis, and interpretation of the data; and in writing the manuscript. The findings and conclusions in this report are those of the authors and do not represent the official position of the funders.

## Results

### Participant demographic and household characteristics

**Fig. 2:**
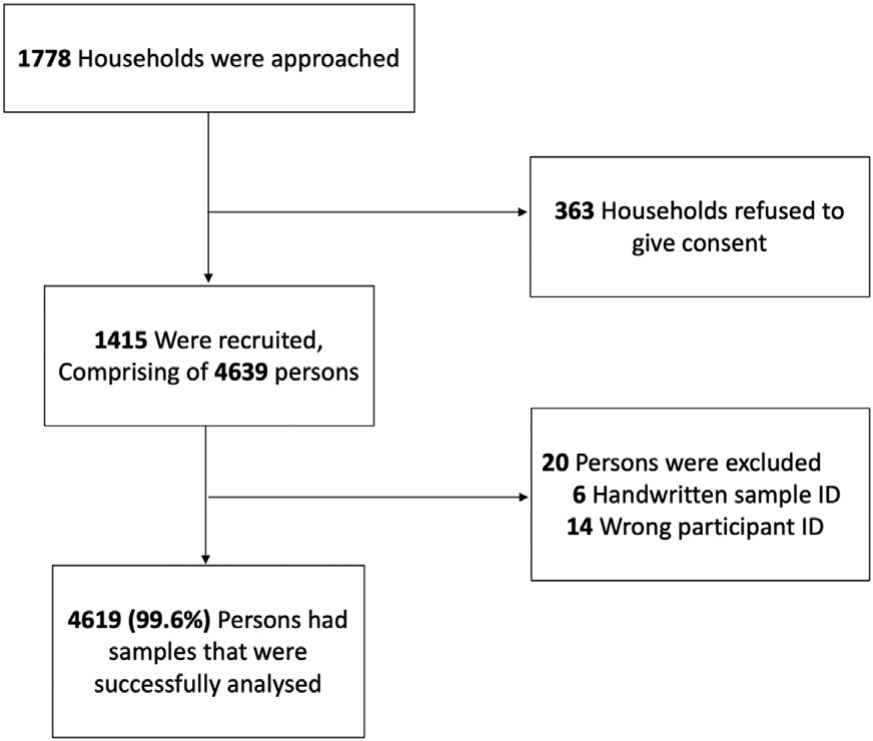
**Survey participant recruitment.** The survey was conducted between 27th December 2021 and 17th January 2022. The survey included primary sampling units (PSU), which were selected with probabilities proportionate to the number of households based on the 2018 census. The second-stage sampling units were selected from lists of households sampled PSUs, then a random systematic sample of households was selected from each PSU within each health zone to the extent feasible.

We enrolled 4639 participants from 1415 households, with 4619 samples from the participants analysed and the remaining 20 samples were excluded due to errors in participant IDs (Fig. 2). This constituted 74% of the target sample of 6248 persons, but a 99.6% (1415/1420) success rate at a household level. The response rate was generally high across all districts however reasons for refusal included cultural and religious beliefs against drawing of blood, mistaking the serosurvey for COVID-19 vaccination programme, lack of incentive and general fear towards COVID-19 related activities. Demographic and household characteristics, comorbidities, participant-reported HIV status, and COVID-19 vaccination status are shown in Table 1, Table 2. In brief, 60% (2751/4619) of the participants were between the ages of 18–50 years, 57% (2617/4619) were female, and 7.8% (361/4619) reportedly had had a chronic illness other than HIV infection.

**Table 1:** Clinical characteristics.

Characteristic	N = 4619^a^
**Sex**	
Female	2617 (57%)
Male	2002 (43%)
**Age group**	
<13 yrs	187 (4.4%)
13–17 yrs	230 (5.5%)
18–50 yrs	2751 (65%)
>50 yrs	1043 (25%)
Unknown	408
**COVID-19 Vaccination status**	
0 doses	3606 (78%)
1 dose	521 (11%)
2 doses	492 (11%)
**SARS-CoV-2 serostatus**	
Negative	865 (19%)
Positive	3754 (81%)
**Comorbidities**	
Yes	361 (7.8%)
No	4258 (92%)
**HIV status**	
HIV–	4457 (96%)
HIV+	162 (3.5%)

a n (%).

**Table 2:** **Socio-demographic characteristics**.

Characteristic	N = 4619^a^
**Occupation**	
Irregular	522 (11%)
Regular	364 (7.9%)
Unwaged	3733 (81%)
**Education level**	
Primary	2086 (45%)
Secondary	1038 (22%)
Tertiary	232 (5.0%)
No formal education	1263 (27%)
**Economic zone**	
Urban	1373 (30%)
Rural	1156 (25%)
bOther	2090 (45%)
**Location**	
Blantyre	768 (17%)
Dedza	527 (11%)
Dowa	441 (9.5%)
Kasungu	645 (14%)
Lilongwe	1327 (29%)
Machinga	477 (10%)
Zomba	434 (9.4%)

a n (%).

b Includes other districts apart from city-harboring districts of Lilongwe, Blantyre and Zomba.

### SARS-CoV-2 seroprevalence estimates

**Fig. 3:**
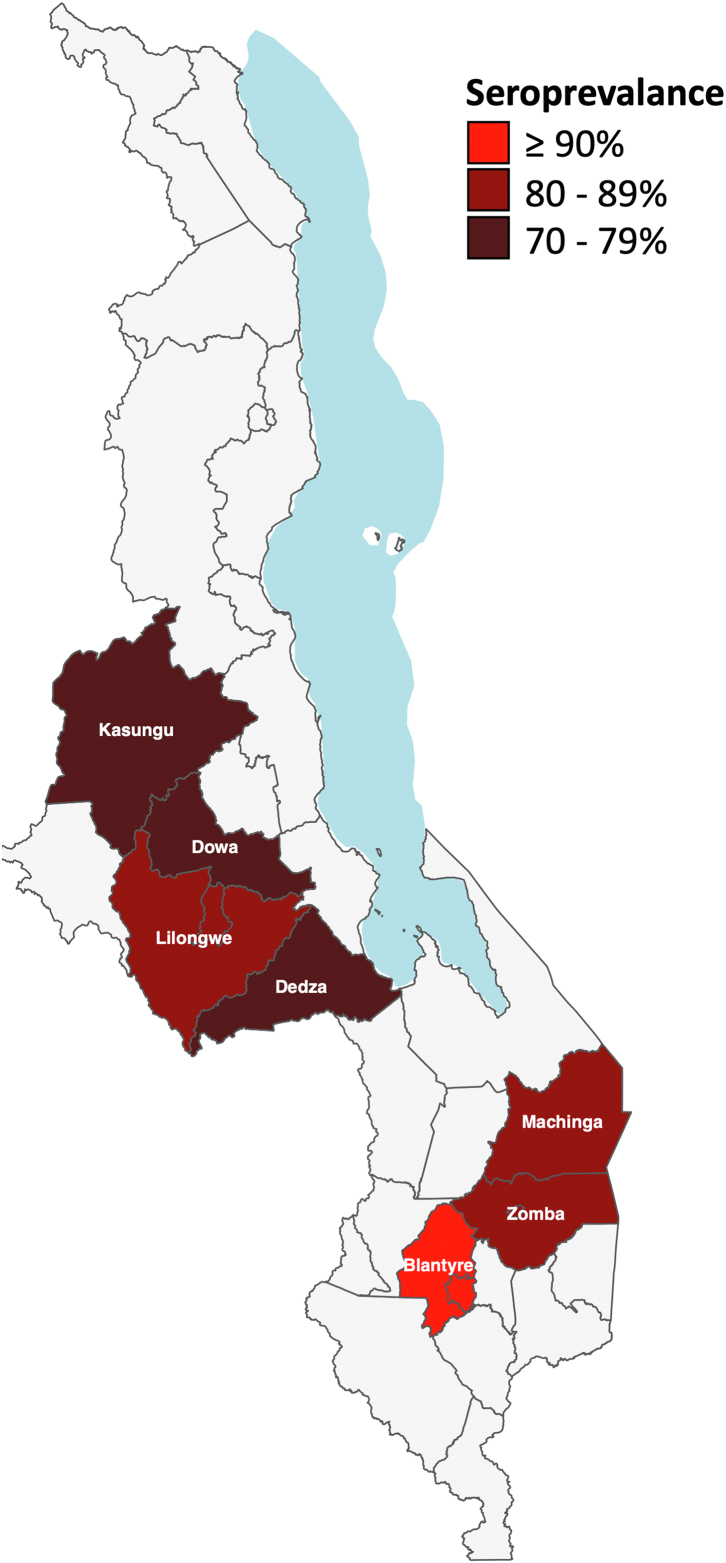
**SARS-CoV-2 seroprevalance by geographical location**.

Among all participants, the overall assay sensitivity and specificity adjusted seroprevalence was 83.7% (95% confidence interval (CI), 79.3%–93.4%). The adjusted seroprevalence was heterogeneous across the districts, ranging from 77.4% (95% CI, 71.1%–88.1%) in Kasungu, to 91.1% (95% CI, 86.1%–98.3%) in Blantyre (Table 3 and Fig. 3).

**Table 3:** SARS-CoV-2 antibody seroprevalence estimates.

	n	Seroprevalence	95% Confidence Interval	Seroprevalence a(adjusted)	95% Confidence Interval a(adjusted)
All	3755/4619	81.3%	80.1%–82.4%	83.7%	79.3%–93.4%
<13 years	124/188	66.0%	59.1%–73.0%	67.4%	57.9%–79.4%
13–17 years	179/230	77.8%	71.9%–83.0%	80.0%	71.6%–91.5%
18–50 years	2261/2750	82.2%	80.7%–83.6%	84.7%	80.1%–94.2%
>50 years	847/1043	81.2%	78.7%–83.5%	83.6%	78.3%–93.6%
Blantyre	678/768	88.3%	85.8%–90.5%	91.1%	86.1%–98.3%
Dedza	406/527	77.0%	73.2%–80.6%	79.2%	72.6%–89.9%
Dowa	334/441	75.7%	75.7%–79.7%	77.8%	70.9%–88.9%
Kasungu	486/645	75.3%	71.8%–78.6%	77.4%	71.1%–88.1%
Lilongwe	1132/1327	85.3%	83.3%–87.2%	87.9%	83.1%–96.6%
Machinga	380/477	79.7%	75.8%–83.2%	81.9%	75.3%–92.6%
Zomba	338/434	77.9%	73.7%–81.7%	80.1%	73.3%–91.0%
Urban	1252/1373	91.2%	89.6%–92.6%	94.2%	90.0%–99.1%
Rural	896/1156	77.5%	75.0%–79.9%	79.7%	74.2%–90.0%

a The sensitivity and specificity of the assay as independently validated in a US study are 96.7% [95% CI 85.5–99.9] and 97.5% [95% CI 92.5–99.9], respectively.

Participants residing in urban areas were more likely to test seropositive than those from rural areas (91% vs. 78%; adjusted odds ratio (aOR) 2.76 [95% CI, 2.16–3.55]) (Table 4). Males were less likely to be seropositive than females (78% vs. 84%; aOR 0.61 [95% CI, 0.52–0.71]). The seroprevalence among the age groups did not vary greatly among those aged 13 years and above; but it was lowest in children younger than 13 years of age (66%) and highest among adults 18–50 years of age (82%). Children 13–17 years of age were more likely to be seropositive than children younger than 13 years of age (78% vs. 66%; aOR, 1.78 [95% CI, 1.14–2.80]). Participants who had received a COVID-19 vaccine were more likely to be seropositive than unvaccinated participants (1 dose vs. no dose, 94% vs. 77%; aOR 4.89 [95% CI, 3.43–7.22]; 2 doses vs.` no dose, 97% vs. 77%; aOR 6.62 [95% CI, 4.14–11.3]).

**Table 4:** Factors associated with SARS-CoV-2 antibody seropositivity.

Characteristic	Seropositivity				Univariable				Multivariable		
	Overall, N = 4,619^a^	Negative, N = 865^a^	Positive, N = 3,754^a^	p-value^b^	N	OR^c^	95% CI^c^	p-value	OR^c^	95% CI^c^	p-value
**Age group**				**<0.001**	4619						
<13 yrs	187 (100%)	63 (34%)	124 (66%)			–	–		–	–	
13–17 yrs	230 (100%)	51 (22%)	179 (78%)			1.78	1.16, 2.76	**0.009**	1.78	1.14, 2.80	**0.011**
18–50 yrs	2751 (100%)	490 (18%)	2261 (82%)			2.34	1.70, 3.21	**<0.001**	1.71	1.21, 2.39	**0.002**
>50 yrs	1043 (100%)	196 (19%)	847 (81%)			2.2	1.56, 3.08	**<0.001**	1.58	1.10, 2.27	**0.013**
Unknown						2.68	1.79, 4.02	**<0.001**	1.83	1.20, 2.82	**0.005**
**Sex**				**<0.001**	4619						
Female	2617 (100%)	417 (16%)	2200 (84%)			–	–		–	–	
Male	2002 (100%)	448 (22%)	1554 (78%)			0.66	0.57, 0.76	**<0.001**	0.61	0.52, 0.71	**<0.001**
**Economic zone**				**<0.001**	4619						
Rural	1156 (100%)	260 (22%)	896 (78%)			–	–		–	–	
Urban	1373 (100%)	121 (8.8%)	1252 (91%)			3	2.39, 3.80	**<0.001**	2.76	2.16, 3.55	**<0.001**
dOther	2090 (100%)	484 (23%)	1606 (77%)			0.96	0.81, 1.14	0.7	1.02	0.86, 1.22	0.8
**Education level**				**<0.001**	4619						
Primary	2086 (100%)	463 (22%)	1623 (78%)			–	–		–	–	
Secondary	1038 (100%)	146 (14%)	892 (86%)			1.74	1.43, 2.14	**<0.001**	1.28	1.03, 1.61	**0.028**
Tertiary	232 (100%)	10 (4.3%)	222 (96%)			6.33	3.52, 12.9	**<0.001**	2.9	1.53, 6.13	**0.002**
No formal education	1263 (100%)	246 (19%)	1017 (81%)			1.18	0.99, 1.40	0.062	0.98	0.81, 1.18	0.8
**Occupation**				**<0.001**	4619						
Unwaged	3733 (100%)	768 (21%)	2965 (79%)			–	–		–	–	
Irregular	522 (100%)	73 (14%)	449 (86%)			1.59	1.24, 2.08	**<0.001**	1.15	0.87, 1.53	0.3
Regular	364 (100%)	24 (6.6%)	340 (93%)			3.67	2.46, 5.74	**<0.001**	1.6	1.02, 2.61	**0.049**
**COVID-19 Vaccination status**				**<0.001**	4619						
0 doses	3606 (100%)	816 (23%)	2790 (77%)			–	–		–	–	
1 dose	521 (100%)	32 (6.1%)	489 (94%)			4.47	3.15, 6.57	**<0.001**	4.89	3.43, 7.22	**<0.001**
2 doses	492 (100%)	17 (3.5%)	475 (97%)			8.17	5.17, 13.9	**<0.001**	6.62	4.14, 11.3	**<0.001**

a n (%).

b Pearson's Chi-squared test.

c OR = Odds Ratio, CI = Confidence Interval.

d Includes other districts apart from city-harboring districts of Lilongwe, Blantyre and Zomba.

Participants who reached secondary education (86% vs. 78%; aOR 1.28 [95% CI, 1.03–1.61]) and tertiary education (96% vs. 78%; aOR 2.90 [95% CI, 1.53–6.13]) levels had a higher seroprevalence than participants at primary education level. Participants with a regular waged job were more likely to be seropositive than unwaged participants (93% vs. 79%; aOR 1.60 [95% CI, 1.02–2.61]). On the other hand, when HIV and comorbidities were analysed in relation to seropositivity, no significant associations were found, and they did not have an impact on the regression model, hence they were not included in the final regression analysis (Table 4).

### COVID-19 vaccination

Of the 4619 participants who were included in the data analysis, 3794 of them had known age and were eligible for COVID-19 vaccination at the time of the study being 18 years and older (Table 5). Overall, 23% (878/3794) of the participants had received at least one dose of the COVID-19 vaccine, with 12% (458/3794) receiving 1 dose and 11% (420/3794) received 2 doses. The age group of 51 years and above had a higher proportion of vaccinated individuals than those between 18 and 50 years (33% vs. 19%, p < 0.001). Moreover, the age group of 51 years and above had a higher proportion of vaccinated individuals that received 2 doses than those between the ages of 18–50 years (19% vs. 8%).

**Table 5:** COVID-19 vaccination.

Characteristic	Overall, N = 3794^a^	0 doses, N = 2916^a^	1 dose, N = 458^a^	2 doses, N = 420^a^	p-value^b^
**Age group**					**<0.001**
18–50 yrs	2751 (100%)	2216 (81%)	312 (11%)	223 (8.1%)	
>50 yrs	1043 (100%)	700 (67%)	146 (14%)	197 (19%)	
**Sex**					**0.011**
Female	2176 (100%)	1689 (78%)	274 (13%)	213 (9.8%)	
Male	1618 (100%)	1227 (76%)	184 (11%)	207 (13%)	
**HIV status**					0.6
HIV-	3668 (100%)	2824 (77%)	441 (12%)	403 (11%)	
HIV+	126 (100%)	92 (73%)	17 (13%)	17 (13%)	
**Location**					**<0.001**
Blantyre	611 (100%)	476 (78%)	44 (7.2%)	91 (15%)	
Dedza	468 (100%)	394 (84%)	61 (13%)	13 (2.8%)	
Dowa	308 (100%)	208 (68%)	83 (27%)	17 (5.5%)	
Kasungu	538 (100%)	382 (71%)	105 (20%)	51 (9.5%)	
Lilongwe	1124 (100%)	834 (74%)	109 (9.7%)	181 (16%)	
Machinga	401 (100%)	364 (91%)	14 (3.5%)	23 (5.7%)	
Zomba	344 (100%)	258 (75%)	42 (12%)	44 (13%)	
**Education level**					**<0.001**
Primary	1602 (100%)	1265 (79%)	202 (13%)	135 (8.4%)	
Secondary	910 (100%)	685 (75%)	95 (10%)	130 (14%)	
Tertiary	206 (100%)	128 (62%)	20 (9.7%)	58 (28%)	
No formal education	1076 (100%)	838 (78%)	141 (13%)	97 (9.0%)	
**Occupation**					**<0.001**
Irregular	462 (100%)	368 (80%)	45 (9.7%)	49 (11%)	
Regular	308 (100%)	169 (55%)	31 (10%)	108 (35%)	
Unwaged	3024 (100%)	2379 (79%)	382 (13%)	263 (8.7%)	
**Economic zone**					**<0.001**
Urban	1123 (100%)	855 (76%)	82 (7.3%)	186 (17%)	
Rural	956 (100%)	713 (75%)	113 (12%)	130 (14%)	
cOther	1715 (100%)	1348 (79%)	263 (15%)	104 (6.1%)	

a n (%).

b Pearson's Chi-squared test.

c Includes other districts apart from city-harboring districts of Lilongwe, Blantyre and Zomba.

All major cities of Lilongwe (16%), Blantyre (15%) and Zomba (13%), had a greater proportion of individuals that received two doses of COVID-19 vaccine compared to the other districts (all <9.6%). Moreover, the proportion of 2-dose vaccinated individuals was higher in urban areas than rural areas (17% vs. 14%, p < 0.001). A greater proportion of participants who had gone up to tertiary education level (28%) received two doses of the COVID-19 vaccine, whereas those at primary and secondary level were 8% and 14%, respectively (p < 0.001). The proportion of 2-dose vaccinated people were higher (p < 0.001) among those with a regular wage (35%) compared to unwaged (9%) or those with an irregular wage (11%).

### National COVID-19 epidemiological trends

**Fig. 4:**
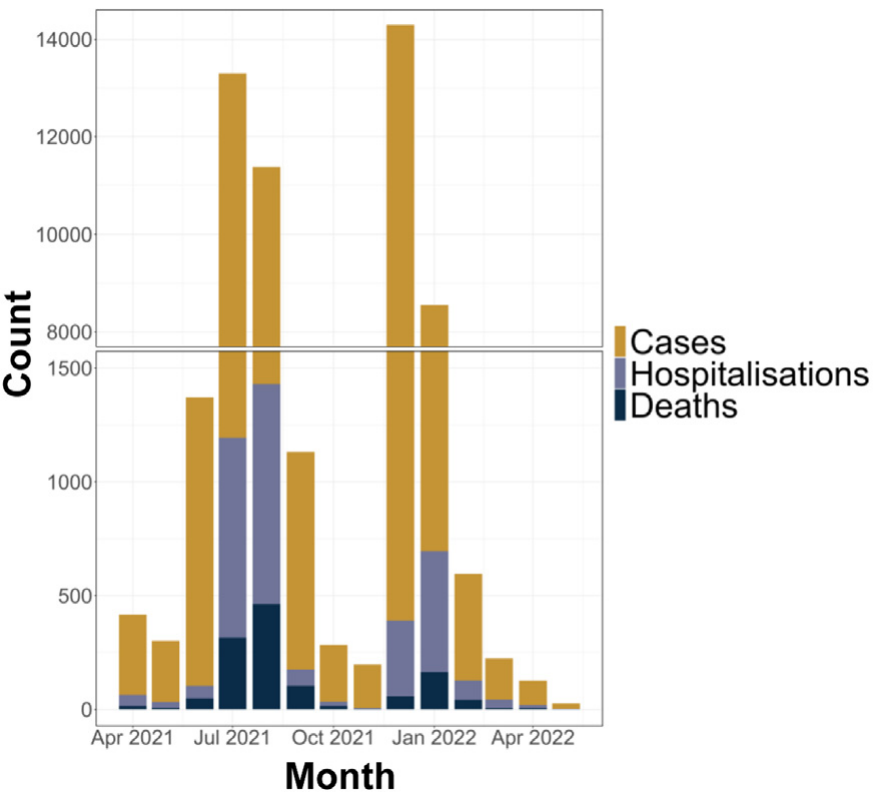
**COVID-19 cases, hospitalisations, and deaths in Malawi, from April 2021 through April 2022.** Shown are incidences of monthly cases, hospitalisations, and deaths attributable to coronavirus disease 2019 (COVID-19). The data were sourced from the Public Health Institute of Malawi (PHIM). Cases included asymptomatic and symptomatic infections with severe acute respiratory syndrome coronavirus 2 (SARS-CoV-2) confirmed by either a nucleic acid amplification assay or a rapid antigen test. Hospitalisations included admissions for SARS-CoV-2 infection, as well as admissions for other illnesses in which SARS-CoV-2 infection was incidentally identified on routine screening at the time of admission. Death attributable to COVID-19 was defined as a death resulting from a clinically compatible illness in a probable or confirmed COVID-19 case unless there is a clear alternative cause of death that cannot be related to COVID disease (e.g., trauma), according to the national guidelines.

Our study was conducted during the fourth wave, which was dominated by the Omicron variant.^21^^,^^22^ During this wave, the daily case incidence increased more rapidly and also decreased more quickly compared to the third (Delta) wave, with the peak in July 2021 and December 2021, respectively (Fig. 4). The number of confirmed clinical cases at the peak of the third wave were 13,300 and during the fourth wave were 14,298, whereas the peak episodes of hospitalisations were 1429 and 695, while deaths were 463 and 164, respectively. The proportion of hospitalisations within the confirmed clinical cases was two-fold lower in the fourth wave compared to the third wave (4.86% (95% CI, 4.52–5.23) (695/14,298) vs. 10.7% (95% CI, 10.2–11.3) (1429/13,330), p < 0.0001). While the proportion of deaths within the confirmed clinical cases was three-fold lower in the fourth wave compared to the third wave (1.15% (95% CI, 1.00–1.34) (164/14,298) vs. 3.48% (95% CI, 3.18–3.81) (463/13,330), p < 0.0001).

## Discussion

The results of our study showed high SARS-CoV-2 seropositivity of 83.7% across the population during the Omicron B.1.1.529 variant-dominated fourth wave. This high seroprevalence was primarily due to prior SARS-CoV-2 infection, as evidenced by the low vaccine uptake in this population and the 77% seroprevalence among participants who had not received a COVID-19 vaccine. Against this background, we observed a reduction in the proportion of severe COVID-19 (hospitalisations and deaths) within reported COVID-19 cases during the Omicron-dominated fourth wave, compared to the Delta-dominated third wave, indicating a dissociation between COVID-19 cases and severe COVID-19. This is in support of Madhi et al. that have observed a similar phenomenon in South Africa, which they termed “decoupling”.^23^

Notwithstanding the reduced number of hospitalisations and deaths in Malawi, the Omicron variant drove a rapid rise in SARS-CoV-2 infections consistent with its high transmissibility^24^^,^^25^ as observed in other settings.^1^^,^^26^ Based on previous estimates from our adult blood donor serosurvey, the estimated seroprevalence by July 2021 was >65%,^10^ reaching around 80% by November 2021 (unpublished), before the Omicron-dominant fourth wave in Malawi, and this is in agreement with other African countries including Kenya,^27^ South Africa^1^ and Nigeria.^28^ In South Africa, a lower rate of hospitalisations and deaths was associated with the high seroprevalence before the onset of the Omicron-dominant wave, driven by both vaccination and infection.^1^ In Malawi, there was at least a two-fold reduction in proportion of COVID-19 hospitalisations and deaths among the clinical cases reported in the fourth compared to third wave. Madhi et al. suggests that, in South Africa, cell-mediated immunity induced through prior infection and vaccination contributed to protective immunity against severe disease.^1^ This suggestion was based on earlier evidence that showed that the Omicron variant highly evades neutralization antibody activity^5^, ^6^, ^7^^,^^29^ but is ably cross-recognized by T cells.^30^^,^^31^ Evidence of T cell-mediated protection against COVID-19 is accumulating and is consistent with their role in viral clearance, where they might not directly prevent infection but halt progression of infection to disease.^32^^,^^33^ However, there still theoretical risk that a more virulent variant could circumvent infection-induced immunity, but this risk would not be biased towards natural immunity alone but also vaccine induced immunity. For now, current evidence supports strong protection against severe COVID-19 from prior infection,^34^^,^^35^ which is even better with hybrid immunity (prior infection plus vaccination).^36^^,^^37^

The SARS-CoV-2 seroprevalence was generally high across all the seven districts surveyed in the current study, with higher seroprevalence in urban compared to rural areas, as reported in our blood donor serosurvey^10^ and in other sub-Saharan African settings.^38^ This is consistent with national COVID-19 data at the time of the study that reported 51% of confirmed cases in the country were from the cities of Blantyre and Lilongwe,^17^ with a caveat that rural areas had relatively fewer SARS-CoV-2 testing sites. The high seroprevalence seen in the urban areas could be attributed to these areas being hotspots for SARS-CoV-2 transmission. Individuals residing in the urban areas were more likely to have attained higher level education and have regular waged jobs, likely increasing their risk of exposure due to increased interaction with others, closed workspaces, frequent travel on public transport and international travel that also increases the risk of exposure to new variants. Supporting this, seroprevalence was higher in those with secondary or tertiary level education, and in those on regular waged jobs. Our study also found men to have a lower seropositivity, which is contrary to male-dominated reported national COVID-19 cases, this could suggest that females experienced less severe forms of COVID-19 (as previously reported^39^) to trigger testing or were less likely to present for testing. Furthermore, consistent with national data showing that the lowest COVID-19 cases were observed in those aged 9 years and below ,^40^ seropositivity was lowest in those aged less than 13 years, but the reasons behind this are still unclear. These findings highlight the need to tailor public health measures according to context as the pandemic progresses.

Our study has some limitations. First, we used an anti-RBD ELISA assay that was developed against the wild type SARS-CoV-2, hence the extensive mutations in the RBD in the Omicron variant could result in false negative results in individuals who were previously or actively infected with this variant. Nevertheless, considering the extremely high seroprevalence, our conclusions are unlikely to be significantly impacted by the theoretical possibility of lower assay specificity. Second, studies have shown that there is potential for cross-reactivity to endemic pathogens which could result in false positives when testing Sub-Saharan Africa serum samples with commercially available ELISA assays.^41^ However, this is very unlikely in our setting, as our earlier published work using the Wantai assay showed minimal cross-reactivity in “pre-pandemic” samples (Jan to March 2020) from Malawi (Seropositivity 0.48% [3/620]), but a high very high seropositivity (>65%) by July 2021.^10^ The first COVID-19 case in Malawi was detected in April 2020. Third, the use of publicly available reported national COVID-19 surveillance data has its own caveats, including under ascertainment of the cases and deaths or changes in testing strategies over time, and this needs to be taken into consideration when interpreting the comparisons across the waves. However, to our knowledge, there were no significant changes in the clinical management policy of severe COVID-19 cases between the third and fourth waves, hence the comparison between the waves is likely accurate. Forth, we had a relatively small numbers of children to give precise estimates of seroprevalence in this population. Finally, the study was conducted and concluded before the end of the fourth wave, hence this could result in seroprevalence estimates being an underestimate of the actual cumulative exposure for Malawi.

The reduction in the proportion of hospitalisations and deaths from COVID-19 during the Omicron-dominant fourth wave in Malawi, in the context of low vaccination coverage and high infection-induced seroprevalence, could be a watershed moment for the COVID-19 pandemic in under-vaccinated countries like Malawi. However, the number of COVID-19 preventable deaths are still significant and a greater focus on identifying and protecting those most at risk is increasingly important. It has been widely reported that the elderly and people with comorbidities are at an increased risk of severe COVID-19,^42^, ^43^, ^44^, ^45^ and in Malawi, a Malawi Government COVID-19 situation report has shown that the median age for those that succumbed to COVID-19 was 61 years with 64.2% being male.^40^ The current vaccination policy for Malawi has expanded the vaccine eligibility age downwards to include those 12 years to 17 years of age for the Pfizer-BioNTech BNT162b2 mRNA vaccine and is also providing booster doses of the same to those that are eligible, but there are not enough vaccine doses to cover the whole eligible but unvaccinated population. Up until a sarbecoviruses or pan-coronavirus vaccine is available, to maximise protection against severe disease in high seroprevalence and resource-constrained settings like Malawi, there might be the need to amend vaccination policy of current COVID-19 vaccines from mass vaccination campaigns to targeted vaccination that focuses on reported at-risk populations including the elderly and those with co-morbidities.

## Contributors

Funding acquisition: KCJ1, ACM, RSH, TDS, KCJ2, NF, ASA, AC, AH, MYRH, MG, Conceptualization of study: ACM, WK, OD, DK, JP, CM, ECB, BC, Methodology: ACM, WK, OD, DK, JP, CM, MK1, ND, Data collection and curation: ULM, DM, KN, KM, Investigation: JM, MM, IC, BO, RK, Formal analysis: KCJ1, ULM, JM, MYRH, Accessed and verified the underlying data: KCJ1, MK1, Project administration: ULM, KN, Writing - original draft: ULM, KCJ1, ACM, Writing - review & editing manuscript writing: ULM, KCJ1, ACM, RSH, TDS, KCJ2, NF, ASA, AC, AH, MYRH, DT, Manuscript review and approval: All authors.

## Data sharing statement

The deidentified data that support the findings of this study are available from the corresponding author upon reasonable request.

## Declaration of interests

All authors declare no conflict of interest.
